# Analyzing the human gut mycobiome – A short guide for beginners

**DOI:** 10.1016/j.csbj.2022.01.008

**Published:** 2022-01-19

**Authors:** Nadja Thielemann, Michaela Herz, Oliver Kurzai, Ronny Martin

**Affiliations:** a Institute for Hygiene and Microbiology, University of Würzburg, Würzburg, Germany; b Research Group Fungal Septomics, Leibniz Institute for Natural Product Research and Infection Biology- Hans Knoell Institute, Jena, Germany; c National Reference Center for Invasive Fungal Infections, Leibniz Institute for Natural Product Research and Infection Biology- Hans Knoell Institute, Jena, Germany

**Keywords:** Mycobiome, Candida, Host-fungal interactions

## Abstract

The human body is a dynamic ecosystem consisting of millions of microbes which are often comprised under the term microbiome. Compared to bacteria, which count for the overwhelming majority of the microbiome, the number of human-associated fungi is small and often underestimated. Nonetheless, they can be found in different host niches such as the gut, the oral cavity and the skin. The fungal community has several potential roles in health and disease of the human host. In this review we will focus on intestinal fungi and their interaction with the host as well as bacteria. We also summarize technical challenges and possible biases researchers must be aware of when conducting mycobiome analysis.

## 1 The mycobiome and its environment

**Fig. 1:**
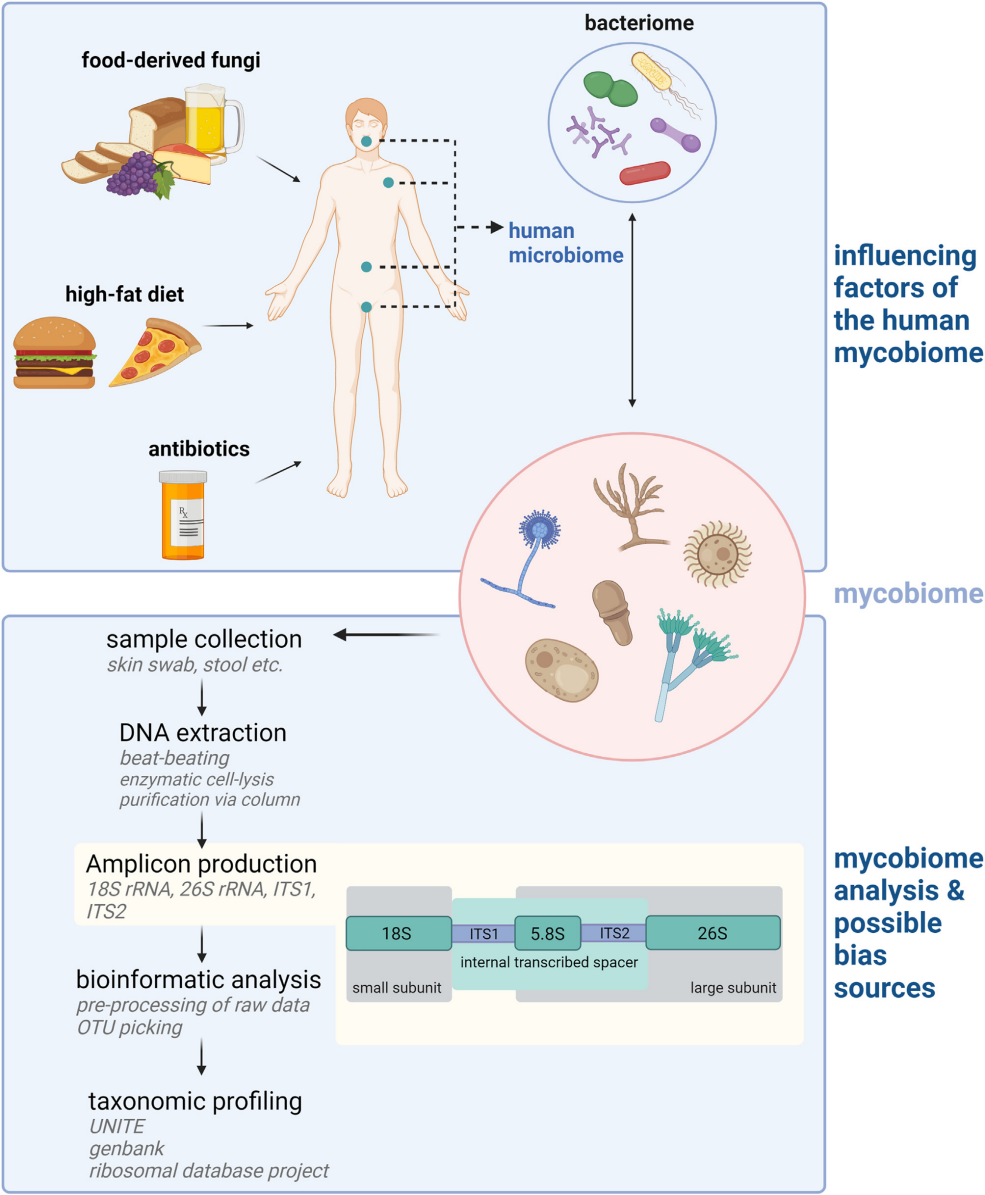
**Influencing factors and possible sources of bias in mycobiota analysis.** The human mycobiome is shaped by different factors like diet, environmental fungi, antibiotic use, and interaction with the resident human bacteriome. For mycobiome analysis, fungal DNA must be extracted from collected samples considering the essential steps for successful fungal DNA extraction. The fungal diversity can be studied with the help of different amplicon production strategies in the fungal rRNA gene locus. Commonly used markers are the 18S as well as the ITS1 & ITS2 regions. The amplified sequences are then processed in bioinformatical analysis, and the taxonomy is assigned due to the comparison of the identified OTUs with available reference sequence databases. The figure was created with BioRender.com.

The term mycobiome defines the fungal part of the microbiome in the human body [1], [2]. Its composition can differ extremely and is influenced by environmental factors such as nutrients, oxygen concentration and pH value. Until now, more than 390 fungal species have been identified in a variety of host niches like the gastrointestinal tract, the skin, the respiratory and the urogenital tract (Fig. 1) [3], [4]. As more than 99% of all intestinal microbial genes are of bacterial origin, fungi account for only a very small amount of the resident gut microbiota [5]. However, they are generally much larger, expose a considerably larger surface to the human host and possess specific routes of interaction with human tissues and the immune system, indicating a distinct role for health or disease of the host [6], [7]. In contrast to the bacteriome, the overall fungal diversity within the human host is relatively low, but more variable between different individuals or even between different samples from the same person [8], [9], [10], [11].

Like the whole microbiome, the mycobiome of infants is highly variable in the first year of life and heavily influenced by birth-mode, breast-feeding practice, diet and increasing age [12], [13], [14], [15]. Recent work indicates that colonization of infants with *Candida* yeasts is already established during birth and breast-feeding [16]. From infancy on, the diet of the human host constantly affects the intestinal microbiome as the human intestine is constantly exposed to several food-borne microbes [17]. Shifts in the intestinal bacteriome are driven by nutrient availability, while the mycobiome is defined by food colonizers and uptake of environmental fungi [18], [19], [20], [21]. Especially the fat and sugar-rich “Western diet”, leading to metabolic changes in the host, is suspected to influence the intestinal microbiota composition and thereby contributes to increasing numbers of chronic diseases like inflammatory bowel disease [19]. Due to these various host-driven influences, a correct distinction between resident and transient food-borne fungi is crucial to characterize the gut mycobiome and maybe to define a “core gut mycobiome” [22].

Especially *Candida* species were identified as key colonizers of the human gut and are supposed to be involved in human health maintenance and disease development [23], [24]. *C. albicans* gut colonization has been identified as the major fungal inducer of human antifungal immunity via Th17 signaling and seems to be regulated by the adaptive host immune system in a similar manner as for bacteria [25], [26], [27]. The mycobiota-induced secretory immunoglobulin A (sIgA) preferentially targets fungal hyphae and thereby regulates fungal commensalism in the human intestine. *C. albicans* colonization in the gut was identified as a potent sIgA inducer and seems to thereby influence its own hyphal morphogenesis to maintain commensalism [28]. Shifts in *Candida* abundance can be linked to multiple disease types such as inflammatory bowel disease, including Crohn’s disease and ulcerative colitis, alcohol-associated liver disease and alcoholic hepatitis [29], [30], [31], [32]. Patients suffering from Crohn’s disease showed increasing levels of antibodies targeting cell wall components of *Saccharomyces* and *Candida* species (Anti-*Saccharomyces cerevisiae* antibodies (ASCA)) in serum samples and an increasing abundance of *Candida tropicalis* and *Debaryomyces hansenii* in the gut [30], [33], [34]. The latter is a food-borne colonizer and might contribute to the development of ulcerative colitis and colorectal cancer [29], [35].

Although *C. albicans* is a frequent colonizer of the human body, it can also cause deadly opportunistic infections. Dissemination of *Candida spp*. from the gut in high-risk patients has been shown to be preceded by alterations of the mycobiome [36]. Additionally, the *C. albicans* peptide toxine Candidalysin triggers a platelet-mediated Th2 and Th17 cell activation which is contributing to a protective allergic response in the lung [37]. Farnesol, a fungal quorum sensing molecule, can modulate the maturation of human dentritic cells [38], [39].

Importantly, intestinal fungi do frequently engage in interactions with other partners in multiple ways and the balance of these interactions affects the human host. These triangle interactions between bacteria, fungi and the human host can be protective or pathogenic or even antagonistic between pathogens [40], [41], [42], [43]. Previous reports showed that virulence of *C. albicans* can be enhanced by the interaction with enterohemorrhagic *Escherichia coli* or reduced by the interaction with *Clostridium difficile* and E*nterococcus faecalis* [44], [45], [46].

## 2 Challenges in gut mycobiome analyses

Past microbiome research was mainly focused on the bacteriome, thus many techniques are not standardized for the study of the intestinal fungi. Therefore, such studies are hampered by non-standardized protocols, technical difficulties, limited availability of reference data and possible biases in data analysis [47]. In the following parts of this review we will address the challenges for gut mycobiome analyses.

## 3 Donor recruitment

As all aspects of human life can affect the gut mycobiome composition, a precise definition of the donor cohort is essential (Fig. 1). Samples must be obtained from donors under standardized conditions. Relevant data on the donor’s lifestyle should be documented as potential confounders. Records should include the overall health state, diet, medical treatment prior to or during the study. Especially the use of antibiotics must be recorded, as they can heavily influence the abundance profile of intestinal fungi [48].

## 4 DNA extraction

Study outcome often depends on the methods used for the recovery of fungal DNA. Direct freezing of samples after collection from human individuals without any further additive is recommended. Addition of RNA stabilizers to fecal samples before freezing can negatively influence the abundance of some fungi as shown for *Penicillium* spec. [11], [49], [50].

If the analysis should include the bacterial and fungal parts of the microbiome, the extraction method must be optimized to obtain the optimal yield and quality of the extracted fungal and bacterial DNA to make sure possible differences are not caused by an extraction bias [51]. Different methods can result in different relative abundances of species like *Penicillium, Malassezia* and *Debaryomyces* [49]. The International Human Microbiota Consortium (IHMC) aimed to optimize and to standardize the bacterial DNA extraction method to enable the comparison of data from different studies by generating the International Human Microbiota Standard (IHMS) Protocols Q (based on the QIAGEN QIAamp DNA Stool kit) and H (non-kit based protocol) [52], [53]. In contrast to bacteria, fungi possess a robust cell wall which is normally composed of chitin, ß-1,3-glucan, ß-1,6-glucan, mannans, several glycoproteins and can also contain components like melanin or a rodlet layer [54], [55], [56]. As fungal DNA extraction relies on efficient cell wall lysis, repeated beat-beating steps followed by enzymatic cell lysis are essential for successful mycobiome analysis from any sample type [50]. The best outcome for combined analysis of mycobiome and bacteriome data from the same samples was achieved by usage of the standardized IHMS Protocol Q with additional repeated beat-beating steps [49], [51]

## 5 Sequencing strategies

The fungal rRNA gene locus is a frequently used target for amplicon sequencing [57]. This region includes the genes for the ribosomal small subunit (18S) and the large subunit (26S) which are separated by the internal transcribed spacer (ITS) regions ITS1 and ITS2 (Fig. 1) [58]. In analogy to the bacterial 16SrRNA gene, the fungal 18S rRNA gene was often used as a target for amplicon production but it seems to be better suited for the discrimination of higher taxonomic ranks [59], [60]. The post-transcriptionally removed ITS regions show a high sequence variability and thus allow a reliable discrimination of the most fungal genera [61], [62]. However, a comparison of ITS1 and ITS2 amplicons showed that commonly used primers identified different fungal species, leading to a different outcome in various studies and thereby influenced the fungal community profile [63], as shown for. *Malassezia* spec. [11], [18], [64]. ITS2 primers showed relatively low bias against specific taxonomic groups, making them a more suitable choice to avoid false-negative results [58]. Primer bias in targeted amplicon sequencing can be circumvented by metagenomic shotgun sequencing approaches. These approaches assess the total DNA from a sample and therefore include bacterial and human DNA. Due to this, the analysis of the mycobiome relies on an accurate filtering of low abundant fungal DNA in these samples, making it more expensive and time-consuming than targeted amplicon sequencing [65].

## 6 Data analysis

Several tools have been developed in the past for the analysis of amplicon or metagenomic data and can clearly influence output and data quality (Table 1) [66], [67]. The key steps in typical data analysis are pre-processing of raw data and operational taxonomic units (OTU) picking followed by taxonomic classification and visualization with statistical analysis (Fig. 1) [4]. Pre-processing of the raw reads and conversion to high-quality output data is crucial and must be carefully conducted as unspecific noise should be reduced but highly conservative filtering could lead to underestimation of specific OTUs [4], [66].

**Table 1:** Overview of frequently used tools for amplicon and metagenomics sequencing data analysis for intestinal mycobiome studies.

Tool	Short Description	Link	Ref.
CONSTAX	Command line tool for improved taxonomy assignment.	Installation via conda package. Documentation: https://constax.readthedocs.io/en/latest/index.html	[94]
Cutadapt	Tool for pre-processing of raw reads which allows trimming of primer and adapter sequences.	https://cutadapt.readthedocs.io/en/stable/	[69]
DADA2	Pre-processing of reads obtained in ITS amplicon sequencing with implemented sequencing error modelling and correction.	https://github.com/benjjneb/dada2	[70]
DAnIEL	Web server-based pipeline for fungal ITS amplicon sequencing data analysis, which allows data analysis, visualisation & statistical analysis as well as comparison of obtained data to publicly available datasets.	https://sbi.hki-jena/daniel	[93]
FastQC	Tool for quality control check of raw reads which allows for monitoring of sequencing errors.	https://www.bioinformatics.babraham.ac.uk/projects/fastqc/	[72]
FindFungi	Pipeline for fungal sequence identification in metagenome datasets.	https://github.com/GiantSpaceRobot/FindFungi	[91]
LEfSe	Algorithm for statistical analysis, linear modelling and visualisation of mycobiome data (OTUs).	https://github.com/SegataLab/lefse	[92]
LotuS2	Pipeline designed for 16S, 18S & ITS amplicon analysis with implemented quality filter.	http://lotus2.earlham.ac.uk/	[88]
mothur	Pipeline originally designed for analysis of 16S rRNA amplicon data, but it is also suitable for ITS amplicon analysis.	https://mothur.org/	[86]
PipeCraft	Flexible pipeline with graphical user interface for analysis of 16S, 18S and ITS amplicon sequencing data.	Available via PlutoF system: https://plutof.ut.ee/#/datacite/10.15156%2FBIO%2F587450	[89]
PIPITS	Pipeline designed for ITS amplicon analysis.	https://sourceforge.net/projects/pipits/	[90]
QIIME 2	Pipeline originally designed for analysis of 16S rRNA amplicon data but also suitable for ITS amplicon analysis.	https://qiime2.org/	[87]
UNITE	Reference database for sequence-based identification of fungi.	https://unite.ut.ee/	[85]
VSEARCH	Pre-processing of reads obtained in metagenomics sequencing.	https://github.com/torognes/vsearch	[71]

The bioinformatic analysis of raw ITS amplicon data is influenced by primer bias, sparsely annotated fungal databases and cannot simply rely on tools established for 16S rRNA data analysis due to the highly variable fungal ITS region [60]. For the analysis of shotgun metagenomics datasets extensive filtering is needed to exclude human or bacterial sequences and identify the rare fungal sequences [68].

The pre-processing of raw reads includes filtering of read length, denoising (e.g. removal of sequencing errors), removal of chimera and singletons/doubletons as well as quality filtering [4]. Several tools have been developed for this pre-processing like Cutadapt for adapter & primer sequence trimming or DADA2 which allows for amplicon error correction (Table 1) [69], [70]. However, most tools were developed for ITS amplicon analysis but some can also be used for metagenomic data sets like VSEARCH [71]. Read quality should be cautiously checked in each approach by tools like FastQC to avoid error accumulation [72].

After pre-processing reads are clustered into OTUs with the help of reference-based and non-reference-based methods. For the closed reference approach, reads are aligned to a reference database and grouped into OTUs based on best match values of the pairwise alignment. *De novo* OTU picking is characterized by clustering of reads against each other without an external database. The open reference approach is a combination of closed reference and de novo approaches, therefor reads are first clustered with the help of an external database and afterwards remaining reads undergo the *de novo* approach [73].

For ITS amplicon sequencing the closed reference approach might be the best choice as comparative classification of an ITS mock community with different pipeline strategies clearly showed improved taxonomic classification for this approach [74].

Mycobiome sequencing data analysis must deal with sparsely annotated reference databases and the question of fungal taxonomy. Different names for the same fungus are commonly in use and can lead to confusion [75]. Additionally, some fungal genera such as *Candida* are not monophyletic. Some medical important species like *C. albicans*, belong to a clade within the Saccharomycetales which is characterized by an alternative translation of the CTG codon [76], [77], [78]. This clade includes however also species which are no longer called *Candida* such as *Clavispora lusitaniae* (formerly *Candida lusitaniae*) and *Meyerozyma guilliermonidii* (formerly *Candida guilliermondii*) [78]. In contrast, other prominent “*Candida”* pathogens like *C. glabrata*, *Pichia kudriazevii* (formerly *Candida krusei*) and *Kluyveromyces marxianus* (*Candida kefyr*) are not part of this clade, were partially renamed and regrouped into other genera [79], [80], [81]. Well curated, high quality databases are essential for a reliable taxonomic classification [82], [83]. Therefore, widely used databases like UNITE are constantly updated, e.g. by implementation of the ISHAM-ITS reference databases (Table 1) [84], [85].

For the data analysis several pipelines including multiple worksteps and analysis tools were generated. Pipelines designed for 16S rRNA analysis like QIIME2 & mother can be used for ITS amplicon analysis but need to be carefully treated as ITS region shows higher variability than the 16S rRNA region and therefore the error potential is elevated [86], [87]. Specific pipelines generated for ITS amplicon analysis like LotuS, PipeCraft & PIPITS circumvent this aforementioned problem and clearly outperform the pipelines with 16S rRNA data analysis origin [67], [88], [89], [90] For metagenomic datasets pipelines like FindFungi enable sequence classification and due to specific false-positive curation they are highly sensitive and specific [91]. Although multiple filters and optimizations are included in these pipelines, errors originating from sample preparation and sequencing cannot be completely removed [60], [67].

For evaluation of mycobiome profiles based on the obtained OTU clusters, data can be examined by e.g. Shannon-index calculation for alpha-diversity measurement and visualization by principle coordinates analysis (PCoA) plots for evaluation of beta-diversity [4]. Several tools like LEfSe enable statistical analysis of the datasets for linear modelling or differential abundance analysis combined with visualization of the data [92]. Recently, the web server DAnIEL has been developed, which includes all steps of ITS amplicon sequencing analysis. Therefor it not only allows data analysis, visualization and extensive statistical analysis but also comparison of the obtained results to publicly available datasets (Table 1) [93]. Extensive pipelines or web servers like this could help to standardize bioinformatics analysis and reduce bias resulting from varying workflows. However, a crucial point in data analysis still is the taxonomic assignment, which should always be checked by multiple tools like e.g. CONSTAX to improve predictions [94].

## 7 Conclusions

Recent studies of the intestinal mycobiome revealed a complex network of fungal, bacterial and human cell interactions. This network has an important influence on the balance between health and disease of the human host. A further standardization of fungal DNA isolation, sequencing methods and bioinformatics data analysis will definitively ease the comprehensive analysis of mycobiome data. A continuous problem for gut mycobiome analyses is the inter- and intraindividual variability. More longitudinal studies will help to characterize stable fungal colonizers in the gastrointestinal tract and to define the resident and transient mycobiome [22], [95]. As illustrated by novel findings for *C. albicans* and *D. hansenii,* frequent gut colonizers might play a crucial role in the development of human disease, inflammation and systemic immune regulation. Such results will improve our knowledge of host-fungus-interactions and might help to develop new therapeutic approaches in the future.

## Author statement

Each named author has substantially contributed to conducting the underlying research and drafting this manuscript. Additionally, the named authors have declared no conflict of interest, financial or otherwise. All authors approved the submission to CSBJ. The manuscript has not been submitted to another journal prior to this submission.

## Declaration of Competing Interest

The authors declare that they have no known competing financial interests or personal relationships that could have appeared to influence the work reported in this paper.
